# Central Dental Association of Northern New Jersey

**Published:** 1884-12

**Authors:** 


### CENTRAL DENTAL ASSOCIATION OF NORTHERN NEW JERSEY.

Meeting at the Residence of Dr. C. S. Stockton, Newark, Tuesday Evening, October 20, 1884.

The President and Vice-President both being absent, Dr. J. Allen Osmun was elected chairman.

After the transaction of the usual routine business, Dr. Edwin T. Darby, of Philadelphia, read a paper upon "Professional Services and Professional Fees." (See page 653.)

### DISCUSSION.

*Dr. J. W. Clowes*—The paper is certainly a very interesting one. There are many points in it that are well worth consideration. As regards fees, I have the matter very well regulated now, so that I quite enjoy the practice of dentistry, with the making out of bills and receiving the pay. When a patient comes to me I say: "I understand that you have come to have your teeth saved. This I cannot do except by doing everything there is to be done; being thorough, doing it well, and doing it all." When they thoroughly understand that, they see the reason for the possibly high figures in the bill. If there is much to do the total will be larger than if there is but little. The charges are moderate, individually, but if there is much work done the aggregate will necessarily be large. As regards charging by the hour, I must say that I do not like it. It is bringing our profession down to the level of a day laborer.

*Dr. C. F. W. Bödecker*—While in Europe, this last summer, I observed a good many hundreds of teeth, and learned something with respect to prices of fillings. I went into the office of a dentist, in a small town in the northern part of Germany, and in the course of conversation he said: "I have to work pretty hard; my fees are very small, and in order to make twenty-five marks (about six dollars), I must have a good many patients." I asked him how much he charged for a gold filling, and he replied that he did not insert any, not being able to get a paying price for them. "But," I said, "in case anybody should come and offer you your price, and asked to have a gold filling inserted?" "Well," he said, "nobody ever will come; or if they do come they say: 'I should like to have a gold filling, but they all tell me that if I do it will fall out, and that a black filling will stay; so I would rather have a black filling;' and the consequence is we put in amalgam fillings altogether, whether the cavity is in front or back." "How much do you charge for amalgam fillings?" "One mark." (About twenty-five cents.) "And what is your price for gold fillings?" "We don't get more than two marks." He told me he bought an eighth of an ounce of gold when he began practice, eight years previously, and three-quarters of it was on hand yet. He had more to do in making artificial teeth than in filling the natural teeth, as the people in that part of the country believed that filling did not do any good, unless they went to an American dentist; the Americans had some funny way of getting around the patients, and could induce them to have gold fillings put in. At all events, he believed it was not so much the skill of the American dentists, as their manner of persuading the patient to have it done, that made them more successful. He told me he was pretty sure he could put in a gold filling just as well as I could, if he could only get the opportunity and the price for it; but the difficulty was that the people would not pay for it, and so he let the gold lie in the drawer. The fees in the larger cities of Europe were a little better. I met some American gentlemen there who charged for the smallest service fifteen marks; and those gentlemen, when they have a cavity of more than ordinary size, call it two, or three cavities, and double or triple the price. I was very much astonished to see that in that way some of the dentists in Europe get quite as much money out of their patients as we do in America, and in some instances a great deal more. On the other hand, German dentists are very moderate in their charges, although some of them are very skillful; they do excellent work, yet they cannot obtain reasonable fees. One gentleman, of whom you have heard, who does almost nothing else than putting in gold fillings, and who inserts a great many of them, cannot get more for a gold filling than about ten to fifteen marks, or two and a half to three dollars; or four dollars for the largest fillings. In the same city there is a gentleman who was for some time in America, and although he is not a very good operator—I have seen several of his patients in that city—he is able to obtain much higher fees than the other dentist, simply because he has been in America. So it seems that an American name draws, so far as dentistry is concerned.

*Dr. E. Parmly Brown*—I do not believe in working by the hour, nor in the English system of charging, as related in the paper. There never yet was created a dentist so good and so godly but that he would give a short hour to his patients if he were to adopt that system of charging. No man is so perfect that he can afford to lead himself into temptation, and no man can remain honest unless he has correct habits, and surrounding circumstances that tend to keep him so.

*Dr. C. E. Francis*—I can remember the time when the average compensation for tin fillings was seventy-five cents, and a dollar and a half for gold. First-class dentists received about double that amount, and three dollars was about the maximum charge of such men as Dr. Parmly, who once remarked to me that one of the most difficult things he ever undertook was to raise his fee for gold fillings from three dollars to five. As to charging by the hour, it does not, to me, look professional.

*Dr. M. L. Rhein*—I have in my practice followed a combination of the two systems of charging. When a patient has a long sitting, I have an average price by the hour for that sitting. For brief operations I make a specific charge.

*Dr. C. S. Stockton*—The only point that I wish to emphasize is, that if the patient of another dentist comes to us and complains of his charges, we should assume that the dentist has been conscientious. We do not know just how much labor may have been expended, and we should stand by each other and our profession, and not criticise the fees of another whose time may possibly be more valuable than our own.

